# Blood oxygen transport and depletion in diving emperor penguins

**DOI:** 10.1242/jeb.246832

**Published:** 2024-03-18

**Authors:** Paul J. Ponganis, Cassondra L. Williams, Jessica M. Kendall-Bar

**Affiliations:** ^1^ Center for Marine Biotechnology & Biomedicine, Scripps Institution of Oceanography, University of California San Diego, La Jolla, CA 92093-0204, USA; ^2^ National Marine Mammal Foundation, 2240 Shelter Island Drive, San Diego, CA 92106, USA

**Keywords:** Arterio-venous shunt, Arterial, Hemoglobin saturation, Vasoconstriction, Venous

## Abstract

Oxygen store management underlies dive performance and is dependent on the slow heart rate and peripheral vasoconstriction of the dive response to control tissue blood flow and oxygen uptake. Prior research has revealed two major patterns of muscle myoglobin saturation profiles during dives of emperor penguins. In Type A profiles, myoglobin desaturated rapidly, consistent with minimal muscle blood flow and low tissue oxygen uptake. Type B profiles, with fluctuating and slower declines in myoglobin saturation, were consistent with variable tissue blood flow patterns and tissue oxygen uptake during dives. We examined arterial and venous blood oxygen profiles to evaluate blood oxygen extraction and found two primary patterns of venous hemoglobin desaturation that complemented corresponding myoglobin saturation profiles. Type A venous profiles had a hemoglobin saturation that (a) increased/plateaued for most of a dive's duration, (b) only declined during the latter stages of ascent, and (c) often became arterialized [arterio-venous (a-v) shunting]. In Type B venous profiles, variable but progressive hemoglobin desaturation profiles were interrupted by inflections in the profile that were consistent with fluctuating tissue blood flow and oxygen uptake. End-of-dive saturation of arterial and Type A venous hemoglobin saturation profiles were not significantly different, but did differ from those of Type B venous profiles. These findings provide further support that the dive response of emperor penguins is a spectrum of cardiac and vascular components (including a-v shunting) that are dependent on the nature and demands of a given dive and even of a given segment of a dive.

## Teaser

**Summary:** Venous hemoglobin saturation profiles reveal a spectrum of cardiovascular dive responses in emperor penguins.

## INTRODUCTION

The management of enhanced oxygen (O_2_) stores in diving mammals and birds underlies their dive capacities, foraging ecology and even their responses to environmental disturbance (Davis, 2014; Ponganis et al., 2011; Williams et al., 2022). Utilization of the respiratory, blood and muscle O_2_ stores during dives depends on multiple factors, including the cardiovascular dive response, lung function at depth, the O_2_–hemoglobin (Hb) dissociation curve, locomotory metabolism in muscle and tissue hypoxemic tolerance (Davis, 2014; Ponganis et al., 2011). Importantly, the decreased heart rate (bradycardia) and peripheral vasoconstriction of the dive response reduce cardiac output and redistribute blood flow, resulting in decreased tissue O_2_ uptake from blood, conservation of blood O_2_ and, in muscle, greater dependency of aerobic metabolism on myoglobin-bound O_2_ (Irving et al., 1941; Scholander, 1940; Scholander et al., 1942).

The intensity of the reduction in heart rate varies with the nature and demands of a given dive or breath hold; heart rate can be variable, only moderately reduced, and modulated with exercise (Butler, 1988; Davis, 2014; Davis and Williams, 2012; Ponganis et al., 2011; Williams et al., 2015). The effects of these variations in heart rate during dives on peripheral blood flow, blood O_2_ extraction [arterio-venous (a-v) O_2_ differences] and the pattern/rate of myoglobin (Mb) desaturation, although often presumed, have been far less studied. Such investigations are important, however, because the degree, duration and frequency of tissue hypoxia (low O_2_ levels) affect dive performance and are critical to mechanisms of extreme hypoxic tolerance that may potentially have translational application to human pathologies (Allen and Vázquez-Medina, 2019; Geiseler et al., 2016; Ponganis, 2019; Tift and Ponganis, 2019). For all these reasons, we examined arterial and venous O_2_ profiles in diving emperor penguins (*Aptenodytes forsteri*) to address how the dive response regulates blood O_2_ extraction and muscle O_2_ utilization in different types of dives. The emperor penguin is ideal for such investigation because it is the only species among diving birds and mammals in which heart rate responses, arterial/venous Hb saturation profiles and muscle Mb saturation profiles have all been documented during dives (Meir and Ponganis, 2009; Meir et al., 2008; Williams et al., 2011).

**Fig. 1.**
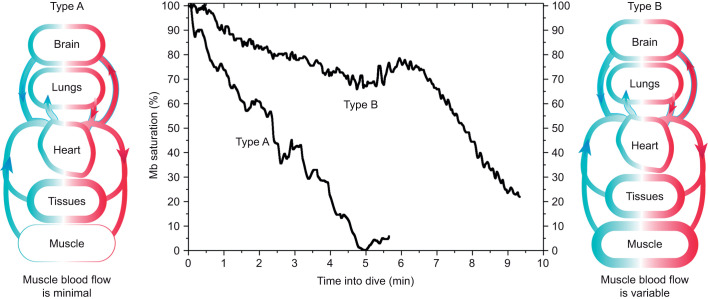
**Representative Type A and Type B myoglobin saturation profiles during dives of emperor penguins.** Type A myoglobin (Mb) saturation profiles were characterized by a rapid decline in saturation consistent with minimal muscle blood flow during the dive. Type B Mb saturation profiles were characterized by a slower, fluctuating decline, and sometimes by a plateau in saturation. Type B Mb saturation profiles were considered the result of variable and intermittent muscle blood flow during dives, including periods of minimal muscle blood flow as evidenced by the more rapid decline in Mb saturation in the final segment of this Type B Mb saturation profile. Data are from Williams et al. (2011).

Our first goal was to determine whether venous Hb saturation profiles could be divided into two types that would be consistent with the two predominant and distinct types of Mb desaturation profiles (Types A and B) previously found in diving emperor penguins (Williams et al., 2011). In Type A dives, Mb desaturated progressively, suggesting minimal to no muscle blood flow (Fig. 1). In contrast, the slower, fluctuating Mb desaturation profiles of Type B dives suggested some continuous but variable and/or intermittent muscle blood flow during dives (Fig. 1). Blood O_2_ extraction by muscle during Type B dives may be more similar to that in flying and running birds, where blood O_2_ extraction and the a-v difference in O_2_ content increase, resulting in a lower venous Hb saturation (Butler et al., 1977; Grubb et al., 1983; Hawkes et al., 2014). Type A Mb desaturation dives (20% of dives) were shorter in duration and less than the 5.6 min aerobic dive limit (ADL, dive duration associated with the onset of post-dive blood lactate accumulation; Ponganis et al., 1997; Williams et al., 2011). Venous Hb saturation profiles that were consistent with Type A and Type B patterns of Mb desaturation would confirm such plasticity in the dive response of emperor penguins.

**Fig. 2.**
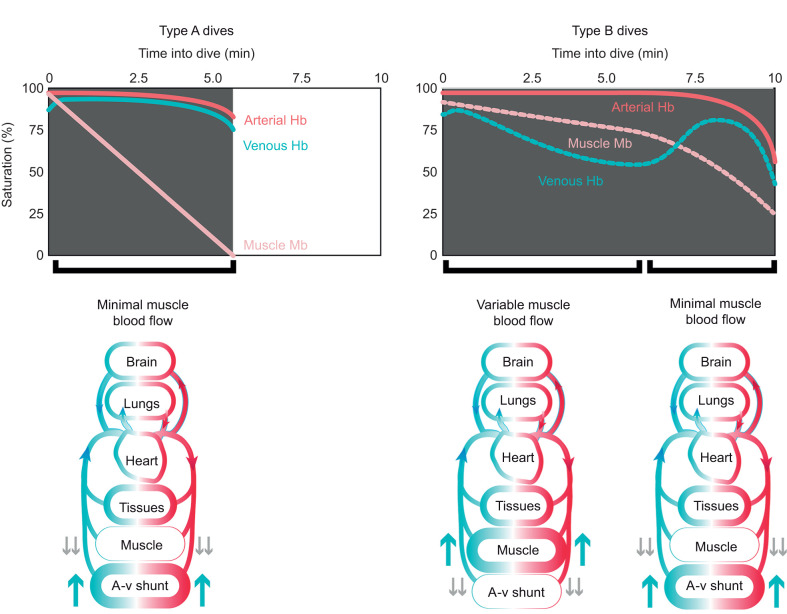
**Hypothetical arterial, venous and muscle saturation profiles and blood flow patterns in dives with a Type A muscle Mb saturation profile and in dives with a Type B muscle Mb saturation profile.** In the Type A dive [minimum muscle blood flow and utilization of an arterio-venous (a-v) shunt], venous saturation remains high during most of the dive, but parallels the decline in arterial saturation during ascent. In contrast, in the Type B dive, maintenance of some, but variable, muscle blood flow allows for blood O_2_ extraction by muscle, a slower decline in muscle saturation, and a decline in venous saturation. However, at about 6–7 min into the dive, a postulated decrease in muscle blood flow and utilization of an a-v shunt allows for a more rapid decline in muscle saturation and an increase in venous saturation during the ascent phase of the dive.

Based on the two types of Mb saturation profiles and prior studies of Hb saturation profiles of emperor penguins, we hypothesized there would be two main patterns of venous Hb desaturation during dives. In one pattern (Type A), venous Hb desaturation would be minimal until the ascent phase of the dive. In the second pattern (Type B), a decline in venous Hb desaturation would predominate throughout the dive. We postulated that in Type A dives, venous Hb saturation would remain elevated because there would be intense vasoconstriction with little to no muscle blood flow and with minimal blood O_2_ extraction by muscle or other tissues during most of the dive (Fig. 2). This pattern is consistent with Type A Mb desaturation profiles (Williams et al., 2011). In addition, in Type A dives, we suspected that a-v shunting could occur and increase venous Hb saturation to near-arterial values. Such arterialized venous blood often occurred during inter-dive surface intervals and even during some dives of these birds (Meir and Ponganis, 2009).

In contrast to Type A venous Hb saturation profiles during dives, we postulated that in other dives (Type B dives), a decline in venous Hb saturation would predominate as a result of maintenance of continuous or intermittent low muscle blood flow, and variable blood O_2_ extraction (Type B venous Hb saturation profile). These patterns are consistent with Type B Mb desaturation profiles (Williams et al., 2011). Although we expected venous Hb saturation would primarily decline in these dives, we also predicted we would find transient fluctuations or interruptions in venous Hb desaturation consistent with the fluctuating declines in Mb saturation observed in muscle during the later segments of Type B saturation profiles. The presence of temporary interruptions in the venous Hb desaturation profiles of Type B dives would provide evidence of (a) transient a-v shunting and/or (b) decreased O_2_ extraction by tissues (secondary to more vasoconstriction and decreased tissue perfusion). Although transient a-v shunting in Type B dives might temporarily elevate venous Hb saturation at any point in the dive, we expected that the venous Hb saturation profile of the entire dive would be dominated by a decline in saturation.

**Fig. 3.**
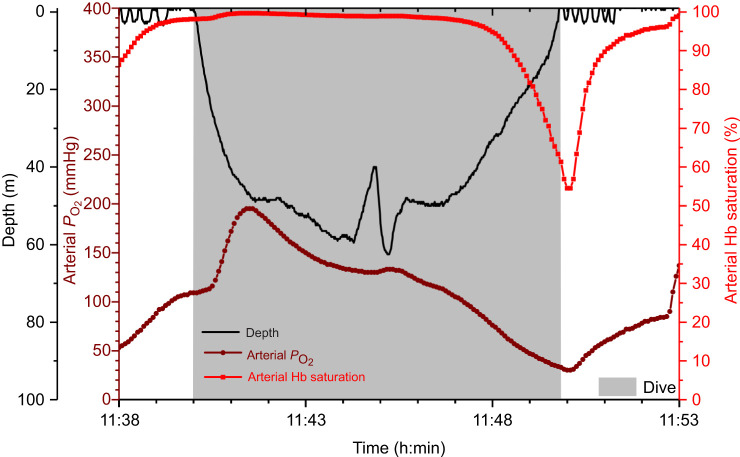
**Arterial partial pressure of O_2_ (*P*_O_2__), arterial hemoglobin saturation and depth profiles of an emperor penguin performing a 9.8** **min, 59** **m dive.** Arterial hemoglobin (Hb) saturation was maintained above 95% until the final 2 min of the dive (the latter half of ascent). Data are for emperor penguin EP1 2008, adapted from Meir and Ponganis (2009).

Our next goal was to evaluate differences between Hb saturation profiles. We analyzed arterial Hb saturation profiles in order to provide a baseline to compare with venous Hb saturation profiles (Fig. 3). We expected that arterial Hb would remain highly saturated during most of the dive because of continued gas exchange and the compressive hyperoxia that occurs in the air sacs and arterial blood of diving penguins (Meir and Ponganis, 2009; Stockard et al., 2005; Williams et al., 2021). Based on our proposed differences in the peripheral dive responses underlying Type A and B venous Hb saturation profiles (Fig. 2), we suspected that Hb saturation in Type A venous profiles would approach and remain near arterial values during much of the dive. Although initial venous Hb saturation of the two types of saturation profiles were not expected to be different, we hypothesized that the increase from start-of-dive to peak venous saturation would be greater in Type A profiles than in Type B and arterial profiles, as we expected a-v shunting to contribute to the arterialization of Type A Hb saturation profiles. We further hypothesized that end-of-dive and mean Hb saturation values would be significantly different between Type A and Type B, with Type A profiles more similar to arterial profiles.

## MATERIALS AND METHODS

Analyses were conducted on data previously collected by this laboratory in 2001, 2003–2005 and 2007–2008 from 15 temporarily captive emperor penguins (*Aptenodytes forsteri* Gray 1844) diving freely at an isolated dive hole in McMurdo Sound, Antarctica (Meir and Ponganis, 2009; Ponganis et al., 2009, 2007). These data included venous O_2_ partial pressure (*P*_O_2__) profiles from 10 penguins and arterial *P*_O_2__ profiles from five penguins (Table S1). Each bird had either venous or arterial data collected; no penguin had both. Body mass ranged from 20 to 30 kg. Arterial and venous Hb saturation profiles were constructed by application of O_2_–Hb dissociation curves to intra-dive arterial and venous *P*_O_2__ profiles obtained with a bio-logging recorder and intravascular *P*_O_2__ electrode (see above references for details). Depth profiles were obtained with an attached time–depth recorder (TDR). Because of recorder memory constraints in the early 2000s, the majority of the *P*_O_2__ data were collected at 15 s intervals; later data were collected at 5 s intervals. Only dives greater than 2 min in duration were included to examine the shape of the Hb saturation profile and the pattern of desaturation. Hb saturation profiles were constructed using the pH 7.5 O_2_–Hb dissociation curve because the change in blood pH during a dive is largely unknown. The typical blood pH of resting emperor penguins is 7.5 (Ponganis et al., 2007); blood pH during the first 3.2 min of dives ranged from 7.35 to 7.47 (Ponganis et al., 2009).

Venous Hb saturation profiles were plotted and dive types identified based on classification criteria. For Type A dives, the classification criteria included (a) venous Hb saturations that increased and/or plateaued for most of a dive and that only declined during the latter stages of ascent, or (b) venous Hb saturations that did not decline below 90% for the entire dive. Type B dives were dominated by progressive declines in venous Hb saturation that were often interrupted by transient increases and or plateaus in Hb saturation that sometimes could even occur at the start of a dive.

Diving behavior parameters, duration and depth, were determined using TDR depth data for all three groups (dives with Type A venous Hb saturation profile, Type B venous Hb saturation profile or arterial Hb saturation profile). Dive duration and depth were compared among the three groups [arterial, Type A (venous), Type B (venous)] to determine any differences in dive characteristics by dive group and to test the hypothesis that dives with Type A Hb saturation profiles were shorter than those with Type B profiles.

The three groups of Hb saturation profiles (arterial, Type A venous and Type B venous) were evaluated and compared. Because dive durations varied and we were examining Hb saturation values at points within a dive, we included fraction of dive duration (how far into a dive the event occurred) to better compare events between dives of different durations. For example, 0.1 fraction of dive time would be 1 min for a 10 min dive or 30 s for a 5 min dive. From these evaluations, we determined the following five saturation values and when they occurred, including the number of minutes into a dive and the fraction of dive duration: (1) start-of-dive, (2) mean, (3) peak, (4) end-of-dive and (5) the magnitude of change between start-of-dive and peak saturation. In addition, we obtained the time into the dive and dive duration fractions until arterial Hb saturation decreased by 2.5%, 5.0% and 10% saturation or the time at which venous Hb saturation decreased by approximately 10% from the peak value. Because depth data were collected at 1 s intervals and the saturation data at 5–15 s intervals, data were often not available precisely at the dive's start or at a given percentage decrease in Hb saturation. Accordingly, the data values closest to the start of a dive and to a given percentage decline from the peak value were used.

Interruptions in the desaturation of Type B Hb saturation profiles were classified as either a positive inflection (increase in saturation >4%) or a plateau (desaturation profile dominated by no change or <4% increase in saturation). The magnitude of change in saturation during an interruption, the time required to reach the peak value of an inflection, and the duration of a plateau in saturation were determined. Depth profile characteristics of the interruption in the dive (descent, ascent, bottom phase, change in vertical direction) were also noted.

### Statistics

We used linear mixed-effects models [packages lme4, lmerTest, MuMin, afex, emmeans and pbkrtest (https://cran.r-project.org/web/packages/nlme/index.html) implemented in R (version 4.2.3; https://www.r-project.org/)] to analyze the data and test hypotheses (https://CRAN.R-project.org/package=MuMIn; http://CRAN.R-project.org/package=lme4; https://CRAN.R-project.org/package=emmeans; https://CRAN.R-project.org/package=afex; Halekoh and Højsgaard, 2014; Kuznetsova et al., 2017). In all models, individual bird was included as a random effect to account for repeated sampling. Including both individual bird and year as random effects typically resulted in a lack of convergence in the model, except for the model examining the effect of depth on duration of all dives. Prior to eliminating complex models that did not converge, control parameters were adjusted using the R package afex to try to resolve convergence issues. However, these adjustments did not fix convergence issues, likely because of the small sample size and the relationship between year and individual penguin (each penguin was only present during one year). Fixed effects included depth, duration and saturation parameters. All models were fitted by maximum likelihood using Akaike's information criterion (AIC). Models were compared using Chi-squared distributed likelihood ratios and, when two models were not significantly different, the simplest model with the lowest AIC value was selected. The best model was compared with a model without the fixed effect to determine whether the fixed effect was significant using Chi-squared distributed likelihood ratios. *P*-values were calculated using Satterthwaite’s method to estimate denominator degrees of freedom for *t*-statistics (Kuznetsova et al., 2017). Marginal and conditional *R*^2^ (*R*^2^_m_ and *R*^2^_c_, respectively) values were obtained to determine goodness of fit (Nakagawa and Schielzeth, 2013). Residuals were visually assessed to confirm model requirements, including normality and homoscedasticity, for all models. Duration, depth and increase in saturation from start to peak values were log-transformed to meet model requirements. Model assumptions were generally met and any minor deviations should not affect statistical results as mixed effect models are known to be robust to violations of model requirements (Schielzeth et al., 2020). Models were used to assess the effect of the three Hb saturation profile groups on duration, depth and saturation parameters. Differences between the three Hb saturation profile groups (arterial, Type A venous, Type B venous) were compared with Tukey's *post hoc* pairwise comparison. Graphics were constructed in Origin (OriginLab, Northampton, MA, USA) or R. Data are expressed as means±s.e.m. Significance was set at *P*<0.05.

## RESULTS

### Hb saturation profiles

**Fig. 4.**
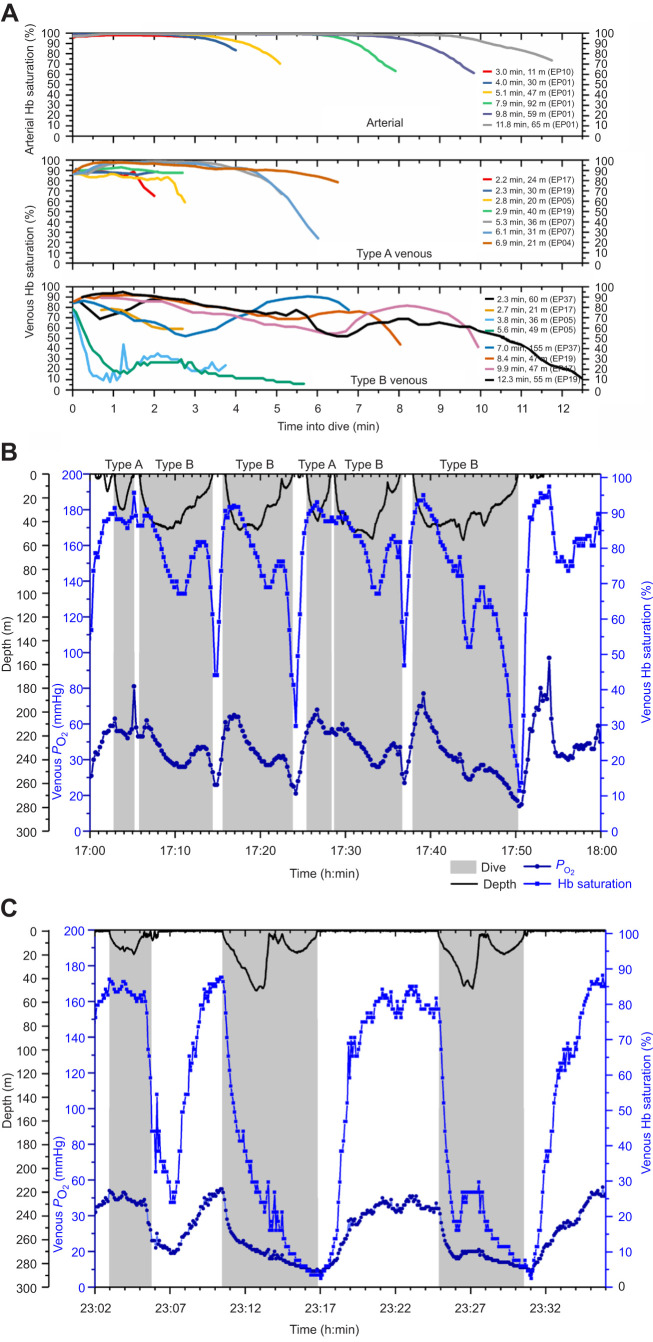
**Hb saturation, *P*_O_2__ and depth profiles for Type A and B venous profiles.** (A) Representative arterial and Type A and B venous Hb saturation profiles from multiple penguins for dives of 2.2–12.3 min duration and 11–155 m depth. Regardless of dive duration or maximum depth, arterial Hb saturation remained above 95% until the ascent phase of the dive. In Type A venous profiles, Hb saturation typically remained elevated during most of the dive (until the ascent). Hb saturation was often arterialized (>90%). In Type B venous profiles, Hb saturation was more varied, often much lower than in Type A dives, and had common interruptions (inflections and plateaus) in the desaturation profile. (B) Venous *P*_O_2__, venous Hb saturation and depth profiles of two dives with a Type A venous profile and three dives with a Type B venous profile (from one penguin, EP19 2004). (C) Venous *P*_O_2__, venous Hb saturation and depth profiles of one dive with a Type A venous profile and two dives with a Type B venous profile (from one penguin, EP05 2003). In B and C, dives are indicated by gray shading.

Venous and arterial Hb saturation profiles were obtained from 102 dives and 64 dives, respectively (Table S1). Review of venous Hb saturation profiles revealed two primary types that met the classification criteria of Type A and Type B (Fig. 4). Of the 102 dives with venous Hb saturation profiles, 31 were classified as Type A and 65 (i.e. over twice as many) were classified as Type B. Six dives which did not fit the criteria were classified as ‘other’ and excluded from further analysis.

### Diving behavior

Mean and median durations for the 31 dives with Type A venous Hb saturation profiles were 4.1±0.35 and 3.1 min, while those for the 65 dives with Type B venous Hb saturation profiles were 9.3±0.49 and 9.0 min (Fig. S1). Mean and median maximum dive depths of dives with Type A Hb saturation profiles were 27±2.2 and 24 m, and those for dives with Type B Hb saturation profiles were 54±2.9 and 51 m. Maximum duration and depth for Type A dives were 9.1 min and 65 m, respectively, and those for Type B dives were 23.1 min and 155 m, respectively. For the 64 dives with arterial Hb saturation profiles, mean and median dive durations were 5.5±0.2 and 5.3 min, respectively. Corresponding mean and median maximum depths were 30±2.2 and 23 m. Maximum dive duration was 11.8 min and maximum depth was 92 m. Regarding the number of dives ≥5.6 min, the ADL, Type A had 8 of 31 dives and Type B had 56 of 65 dives. For dives with arterial profiles, 25 of 64 dives were ≥5.6 min.

For all dives, depth had a significant effect on duration (χ^2^_1_=10.13, *P*<0.01) and explained 43% of variation under the model, whereas bird ID and the interaction between depth and year contributed an additional 19% of explanatory power (*R*^2^_m_=0.434, *R*^2^_c_=0.621).

**Fig. 5.**
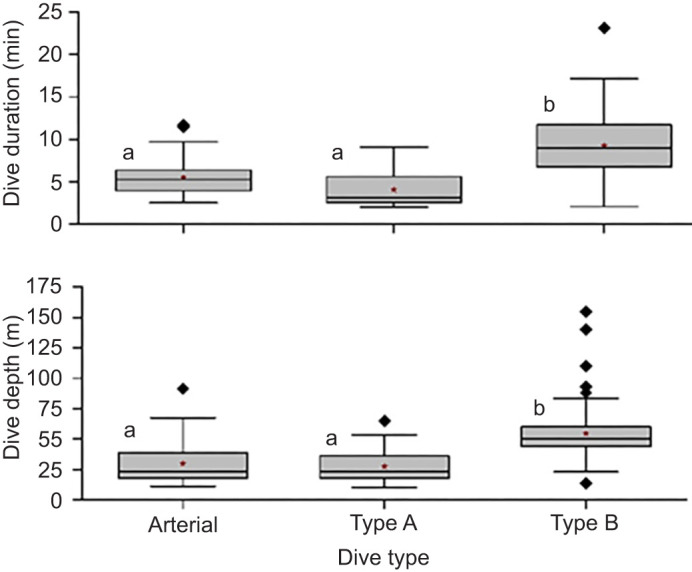
**Box plots of dive duration and maximum dive depth in dives with arterial, Type A venous and Type B venous Hb saturation profiles.** Dives with Type B saturation profiles were significantly longer and deeper (*P*<0.05) than dives with either Type A venous profiles or arterial saturation profiles, but the latter two groups did not differ significantly (Tukey's *post hoc* pairwise comparison). Within each panel, different letters represent a significant difference between the dive groups. *N*=15 penguins, 160 dives for all analyses. Boxes indicate the upper and lower quartiles and include the median line; whiskers extend to 1.5 times the interquartile range and outliers are indicated by diamonds. Mean values are represented by a red star.

In the models assessing the effect of Hb saturation profile group on depth and duration, profile group had a significant effect on both duration (χ^2^_1_=60.34, *P*<0.0001) and depth (χ^2^_1_=41.24, *P*<0.001) (Fig. 5). Profile group explained 38% of variation in duration and 29% of variation in depth under the model, whereas the effect of individual bird contributed an additional 14% and 35% of explanatory power in models for duration and depth, respectively (duration: *R*^2^_m_=0.438, *R*^2^_c_=0.677, depth: *R*^2^_m_=0.291, *R*^2^_c_=0.641). Tukey's *post hoc* pairwise comparison demonstrated that there was no significant difference in depth or duration of dives between the arterial and Type A venous groups, but the dives of the Type B venous group were significantly deeper and longer than dives in the Type A venous group and the arterial group (Fig. 5).

### Analysis of Hb saturation profiles

#### Type A venous Hb saturation profiles

In Type A venous Hb saturation profiles, saturation often increased and then plateaued, remaining elevated throughout most of the dive (Fig. 4). Venous Hb saturation increased by 11.4% to a mean peak value of 94.1% within 1.5 min of the start of dive (0.4±0.04 fraction of dive time) (Table 1). The mean net decrease between peak and end-of-dive saturation in all Type A profiles was 19.6±3.9%. Saturation declined by less than 10% from peak values in 14 of the 31 dives (45%). In the other 17 dives, saturation declined greater than 10% from peak values, reaching 83.3±1.4% by 3.2±0.4 min after the start of dive (0.75±0.05 fraction of dive time), and 1.7±0.3 min after the time of peak saturation. Mean end-of-dive saturation of all Type A dives was 74.5% (Table 1).

**Table 1.**
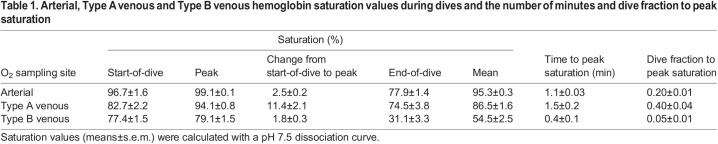
Arterial, Type A venous and Type B venous hemoglobin saturation values during dives and the number of minutes and dive fraction to peak saturation

#### Type B venous Hb saturation profiles

In Type B venous Hb saturation profiles, the profile was dominated by a progressive decline throughout much of the dive (Fig. 4). Type B venous Hb saturation profiles increased by 1.8% to a mean peak value of 79.1% within 0.4 min of the start of a dive (Table 1). Often, the initial saturation was the peak value. In all Type B Hb saturation profiles, saturation decreased ≥10%, reaching a mean net decrease between peak and end-of-dive saturation in the 65 Type B profiles of 48.1±3.1%. Saturation declined from peak values to 68.8±1.7% within 1.5±0.2 min (0.18±0.02 fraction of dive time) of the start of dive, and 1.0±0.1 min after the time of peak saturation. Mean end-of-dive saturation of all Type B profiles was 31.1% (Table 1).

Temporary interruptions in the desaturation profiles occurred in 64 of 65 Type B Hb saturation profiles (e.g. Fig. 4). These interruptions in desaturation were either a positive inflection or a plateau in saturation (see Materials and Methods). Among these 64 dives, 53.8%, 32.3% and 12.3% had one, two or three interruptions in desaturation, respectively, for a total of 103 interruptions; 81.6% of the 103 interruptions in desaturation in the Type B profiles were transient positive inflections. The mean increase in saturation of the inflections was 15.1±9.9% and the mean time to reach peak value was 82.1±49.8 s. Plateaus occurred in the remaining interruptions in the desaturation profiles. The mean range of saturation change and duration of plateaus were 2.4±1.5% and 107.4±56.0 s. Of these interruptions in venous saturation profiles, 66% increased saturation >10%; the maximum increase in saturation was 46.8%. The initial transient inflection in saturation occurred during descent in 48.4% of all Type B dives, during ascent in 28.1%, and at maximum depth in 23.4%. Among the 64 dives with transient interruptions in venous desaturation, 44.6% had two to three such interruptions in the saturation profile. In these 29 dives, eight had a later interruption in desaturation at maximum depth. Consequently, maximum depth was associated with an interruption in venous desaturation in 23 of all 64 dives (35.9%).

#### Arterial Hb saturation profiles

In dives of the arterial Hb saturation profile group, saturation increased after the start of a dive by 2.5% over the first minute to a mean maximum value over 99% (Table 1). Arterial Hb saturation typically remained high (95–100%) throughout most of the dive. Arterial Hb saturation only declined significantly during the final ascent phase of the dive (Fig. 4A). Dive duration had a significant effect on the time into a dive at which 10% desaturation occurred in these profiles (χ^2^_1_=87.24, *P*<0.001; Fig. 4A). Duration explained 79% of variation under the model, whereas the effect of bird contributed an additional 5% of explanatory power (*R*^2^_m_=0.793, *R*^2^_c_=0.846). Arterial Hb saturation decreased to 96.6±0.1% by 3.8±0.2 min into a dive (0.70±0.02 fraction of dive time, *n*=63), to 94.5±0.1% at 4.3±0.2 min into a dive (0.77±0.01 fraction of dive time, *n*=57), and to 89.2±0.1% by 4.8±0.2 min into a dive (0.85±0.01 fraction of dive time, *n*=53). Mean end-of-dive arterial Hb saturation for all dives was 77.9% (Table 1).

#### Comparisons among arterial and venous (Type A and B) Hb saturation profile groups

##### Start-of-dive Hb saturation

In the model assessing the effect of the saturation profile group on start-of-dive Hb saturation, group had a significant effect on saturation (χ^2^_2_=13.81, *P*<0.01; Fig. S2A). Saturation profile group explained 40% of variation in start-of-dive saturation, whereas the effect of individual bird contributed an additional 41% of explanatory power in the model (*R*^2^_m_=0.403, *R*^2^_c_=0.812). Tukey's *post hoc* pairwise comparison demonstrated that there was no significant difference in start-of-dive saturation between dives with Type A and Type B venous Hb saturation profiles (*t*_157.9_=−1.9, *P*=0.46), but start-of-dive arterial Hb saturation values were significantly higher than Type A (*t*_20.2_=−3.6, *P*<0.01) and Type B (*t*_19.2_=3.04, *P*<0.05) venous Hb saturation profiles (Fig. S2A).

##### Magnitude of increase in Hb saturation from start-of-dive to peak saturation

In the model assessing the effect of Hb saturation profile group on the magnitude of saturation increase from start-of-dive to peak Hb saturation, profile group had a significant effect on the magnitude of saturation increase (χ^2^_2_=52.99, *P*<0.0001; Fig. S2C). Profile group explained 34% of variation in the magnitude of increase in saturation, whereas the effect of individual bird contributed only an additional 6% of explanatory power in the model (*R*^2^_m_=0.342, *R*^2^_c_=0.402). Tukey's *post hoc* pairwise comparison demonstrated that there was no significant difference in saturation change (start-of-dive to peak) between arterial and Type B profiles (*t*_18.6_=1.84, *P*=0.18), but the increase in saturation (start-of-dive to peak) of Type A profiles was significantly higher than that in arterial profiles (*t*_26.2_=4.516, *P*<0.001) and Type B profiles (*t*_120.6_=7.911, *P*<0.0001) (Fig. S2C).

##### Mean Hb saturation

In the model assessing the effect of saturation profile group on mean Hb saturation, profile group also had a significant effect on mean Hb saturation (χ^2^_2_=34.23, *P*<0.0001; Fig. S2B). Profile group and duration explained 56% of variation in mean saturation, whereas the effect of individual bird contributed an additional 31% of explanatory power in the model (*R*^2^_m_=0.573, *R*^2^_c_=0.896). Tukey's *post hoc* pairwise comparison demonstrated that there was no significant difference in mean saturation between arterial and Type A profiles (*t*_19.6_=−2.3, *P*=0.08), but mean saturation values of Type B profiles were significantly lower than Type A (*t*_155.3_=5.4, *P*<0.0001) and arterial (*t*_19.5_=4.0, *P*<0.05) mean dive saturation values (Fig. S2C).

##### End-of-dive Hb saturation

In the model assessing the effect of saturation profile group on end-of-dive Hb saturation, profile group had a significant effect on end-of-dive saturation (χ^2^_2_=19.07, *P*<0.0001; Fig. S2D). Profile group and duration explained 67% of variation in end-of-dive saturation, whereas the effect of individual bird contributed an additional 17% of explanatory power in the model (*R*^2^_m_=0.513671, *R*^2^_c_=0.837). Tukey's *post hoc* pairwise comparison demonstrated that there was no significant difference in end-of-dive saturation between arterial and Type A profiles (*t*_20.6_=−1.8, *P*=0.20), but mean saturation of Type B profiles was significantly lower than Type A (*t*_163.3_=3.7, *P*<0.001) and arterial (*t*_20.1_=3.6, *P*<0.01) end-of-dive saturation values (Fig. S2D).

## DISCUSSION

### Dive behavior

Although the dive behaviors of penguins at the experimental isolated dive hole have been described in prior studies (Ponganis et al., 2007, 2001, 2003, 2000), we first briefly review the dive behavior of the birds to support our comparisons of arterial, venous and muscle O_2_ profiles from different dive studies, and to remind readers of the similarities and differences of these dives with those at sea. The dives of these penguins at an isolated dive hole were primarily under 50 m, at the shallow but common end of their diving spectrum (30–70% of all dives at sea) (Kirkwood and Robertson, 1997; Kooyman and Kooyman, 1995; Sato et al., 2011). Most dive durations were less than 12 min; there were 15 dives between 12 and 17 min and one dive of 23.1 min (see Fig. S1). These durations were similar to those at sea. This pattern of diving was associated with the routine hunting and capture of the sub-ice fish *Pagothenia borchgrevinki* (Ponganis et al., 2000). At sea, this fish is a minor prey item (Cherel and Kooyman, 1998; Kirkwood and Robertson, 1997). Dive duration at the isolated dive hole correlated with maximum depth but was highly variable, again similar to the prior studies (Fig. S1). Although most dives at sea are less than the ADL, there was a high percentage of dives in the present study that were greater than the 5.6 min ADL. These longer duration dives may be a result of several factors: (1) at the experimental dive hole, birds must dive under the fast ice to find fish and return to the hole to breathe, whereas at sea, they can surface and breathe without a long return as they typically dive in the pack ice or in open water, (2) as a result of localized depletion of prey fish near the dive hole by a large number of penguins during the season, the birds may have to travel farther underwater to find fish, and (3) unlike the studies at sea (Kooyman and Kooyman, 1995; Sato et al., 2011), dives less than 2 min were not included in the present study; this reduces the number of short duration dives and increases the percentage of longer dives in the present study.

Maximum depths and durations of arterial and venous Type A Hb saturation profile groups were in the same range, but dives in the Type B venous Hb saturation group were deeper and longer. Type A venous Hb desaturation profiles occurred in 32% of all venous profiles, similar to the distribution of Type A and B muscle Mb desaturation dives (Williams et al., 2011). As in the muscle studies, dives with Type A venous profiles were also shorter in duration than those with Type B venous profiles (Fig. 5), and similar in duration to Type A muscle Mb desaturation dives (4.2 min) (Fig. 5) (Williams et al., 2011). The similarities of Type A venous and Type A muscle desaturation dives in dive distribution and dive duration support the hypothesis that the characteristics and intensity of the dive response are the same in both the muscle and venous Type A dives (i.e. little to no muscle blood flow during the dive, primary reliance of muscle metabolism on Mb–O_2_, and limited tissue O_2_ extraction by tissues in these dives).

While it is tempting to speculate as to why some dives are Type A and others are Type B, both the current study and the earlier study by Williams et al. (2011) have fairly small sample sizes. In the present study of venous Hb saturation profiles, 10 birds with 96 dives were represented, with the number of dives per bird ranging from 2 to 19 (Table S1). While Type A dives were shorter and shallower than Type B dives in both studies, the dive profiles were not distinct. Both Type A and Type B dives had intra-dive sub-ice ascents suggestive of foraging for *P. borchevenki*. Further, in the Mb saturation study, there were no Type A dives greater than the ADL (5.6 min); however, in the current study, over a quarter of Type A dives (8 of 31) were equal to or greater than 5.6 min. Finally, there was also not a typical distribution pattern of Type A versus Type B dives. Type A dives were not consistently an initial dive before a series of Type B dives and could occur in a series themselves. Future studies measuring blood O_2_, as well as accelerometry, in a larger number of dives per bird may be able to provide more insight.

### Study limitations

One limitation of our interpretation of venous blood O_2_ profiles is the posterior vena caval location of the O_2_ electrodes. Although we make inferences as to overall patterns of O_2_ depletion and muscle perfusion, the venous *P*_O_2__ and Hb saturation data are not mixed venous values and not necessarily indicative of the entire venous system.

Another limitation in data interpretation is the potential decrease in Hb's affinity for O_2_ due to a lower (more acidic) pH (the Bohr shift) and/or an increase in temperature throughout the course of a dive. Because the time course of any pH changes during dives of emperor penguins is not known, we used the pH 7.5 O_2_–Hb dissociation curve to convert *P*_O_2__ values to Hb saturation. Temperature correction of the dissociation curve of emperor penguins was not possible because, although arterial and vena caval temperatures increase slightly during dives of penguins at the isolated dive hole (Ponganis et al., 2001, 2004, 2003), the temperature sensitivity of their O_2_–Hb dissociation curve is not known. As recently reviewed for the Bohr shift in penguins (Meir and Ponganis, 2009; Signore et al., 2021) and for increased temperature in high-flying geese (Meir and Milsom, 2013), we expect that a decrease in pH or an increase in temperature would decrease Hb's affinity for O_2_ during longer dives, enhancing O_2_ delivery to tissues and lowering venous Hb saturation. However, that does not affect our interpretation of blood flow patterns from Hb saturation profiles during dives.

### Arterial Hb saturation profiles

We begin discussion of Hb saturation profile with the arterial Hb saturation profiles because this analysis is useful for interpreting venous Hb saturation profiles. We also remind readers that penguins were only equipped with one O_2_ electrode (Table S1). Arterial and venous data were not collected simultaneously. Despite compression hyperoxia during descent (Stockard et al., 2005; Williams et al., 2021), the initial increase in arterial Hb saturation was minimal and rapid. This was secondary to the shape of the O_2_–Hb dissociation curve, an adequate arterial *P*_O_2__ and an already high Hb saturation at the start of the dive (Meir and Ponganis, 2009; Ponganis et al., 2009, 2007). As the dive progressed, arterial Hb saturation was well maintained at depth as a result of continued gas exchange and elevated air sac *P*_O_2__ (Figs 3 and 4A). Despite expected decreases in the respiratory O_2_ fraction, arterial, as well as air sac, *P*_O_2__ was still high because of the elevated ambient pressure at depth (Boyle's Law) (Stockard et al., 2005; Williams et al., 2021). The decline in arterial Hb saturation was dependent on dive duration, with the rate of arterial desaturation slowing in longer dives. In the longest (11.8 min) arterial dive, arterial saturation slowly declined, reaching 89.7% at 10.3 min into the dive (Fig. 4A). Arterial Hb saturation did not begin to decline more rapidly until ascent, and especially the latter part of ascent [secondary to a rapid decrease in *P*_O_2__ due to a decreased respiratory O_2_ fraction (Dalton's Law) and the decline in ambient pressure (Boyle's Law); Stockard et al., 2005; Williams et al., 2021].

This type of overall high arterial Hb saturation profile at depth has been reported in diving California sea lions and human breath-hold divers (McDonald and Ponganis, 2012; McKnight et al., 2021; Mulder et al., 2021; Ruesch et al., 2021). The decline in *P*_O_2__ at the end of dives appears uneventful in penguins and pinnipeds (McDonald and Ponganis, 2012; Meir and Ponganis, 2009), but, in humans, this decrease can be associated with shallow water blackout (Lindholm and Lundgren, 2009). In contrast to emperor penguins, sea lions and humans, arterial Hb saturation in elephant seals declined continuously throughout dives after an early initial peak (Meir et al., 2009). This is most probably secondary to start-of-dive air exhalation, early alveolar collapse, and lack of gas exchange at depth in seals (Fahlman et al., 2009; Kooyman et al., 1970, 1971, 1973a). All evidence indicates that gas exchange continues to much deeper depths in penguins and sea lions (Kooyman et al., 1973b; Kooyman and Sinnett, 1982; McDonald and Ponganis, 2012; Ponganis et al., 1999).

The analysis of the emperor penguin's arterial Hb saturation profiles provides two key considerations in the evaluation of venous Hb saturation profiles. First, any large increases in venous Hb saturation during descent and even in other phases of the dive would more likely be secondary to decreased tissue oxygen extraction or to a-v shunts than to the minimal increase observed in arterial Hb saturation. Second, any rapid declines in venous Hb saturation during ascent may be secondary not only to increased tissue O_2_ extraction but also, and perhaps more significantly, to the rapid decline observed in arterial Hb saturation at the end of a dive.

### Venous Hb saturation profiles

#### Type A Hb saturation profiles are consistent with Type A Mb saturation profiles

**Fig. 6.**
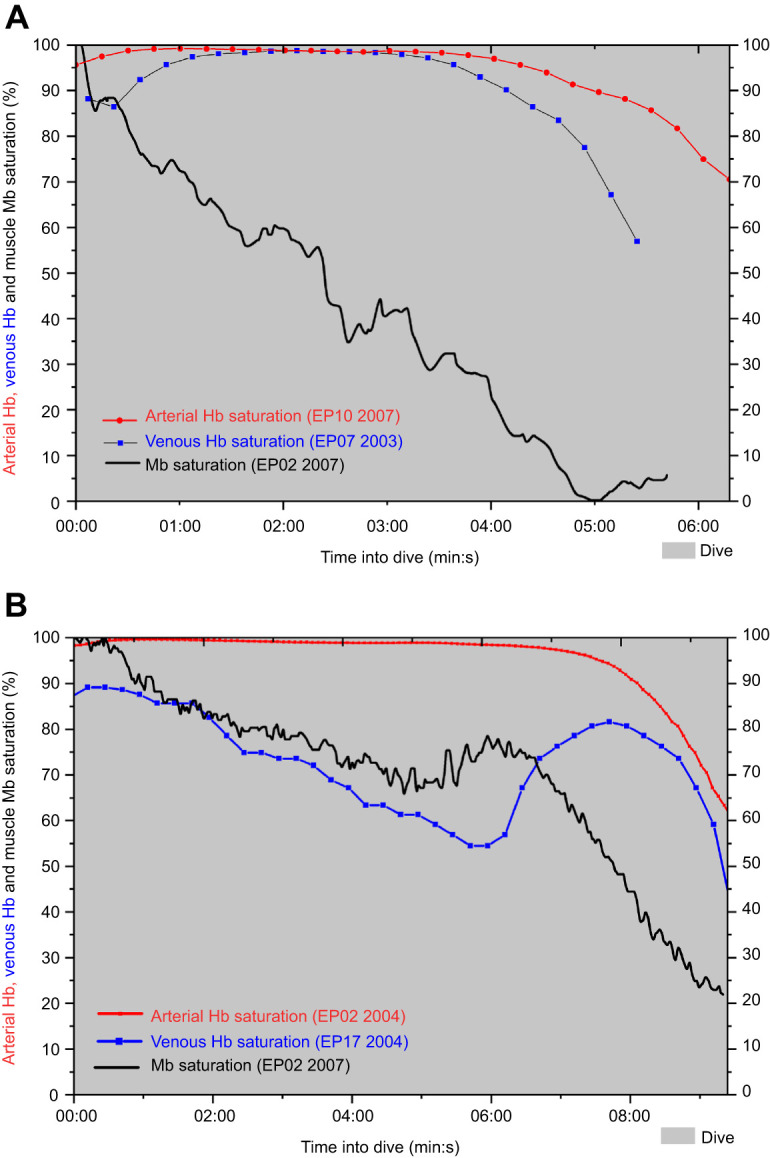
**Composite comparisons of arterial and venous Hb saturation profiles and muscle Mb saturation profiles in shallow dives of similar duration from different penguins.** (A) Type A venous Hb and muscle Mb saturation profiles. (B) Type B venous Hb and muscle Mb saturation profiles. Mb saturation profiles adapted from Williams et al. (2011).

Provided the posterior vena caval Hb saturation is representative of all venous blood, the magnitude and time course of desaturation of muscle Mb and arterial Hb and venous Hb in Type A dives are consistent with the hypothesis of simultaneous a-v shunting and regional vasoconstriction to tissues such as muscle during most of the duration of a dive with a Type A venous Hb (and muscle Mb) saturation profile (Fig. 6A). During the first 4 min of the dive in Fig. 6A, the calculated a-v difference in Hb-bound O_2_ was less than 2.5 ml O_2_ dl^−1^, more than 50% less than the resting value and consistent with this hypothesis of simultaneous a-v shunting and regional vasoconstriction to tissues. The decline in venous Hb saturation towards the end of the dive reflects at least partially the simultaneous decline in arterial Hb saturation during ascent. If a-v shunting decreases during ascent, that decline in venous Hb saturation during ascent may also be secondary to increased tissue perfusion and blood O_2_ uptake by tissues, especially muscle (early re-loading of O_2_-depleted Mb; Thompson and Fedak, 1993). However, the similarities between Type A and arterial end-of-dive Hb saturation values suggest the decline in venous Hb saturation is predominantly a result of the decline an arterial Hb saturation rather than increased O_2_ uptake by muscle, which is again consistent with Type A Mb saturation profiles (Williams et al., 2011).

#### Similarity of Type A and arterial saturation profiles supports a-v shunting

Type A venous Hb saturation profiles, characterized by an increasing saturation or plateau during most of the dive, were more similar to arterial Hb saturation profiles than to Type B Hb saturation profiles (Fig. 4). The start-of-dive saturation was not significantly different between Type A and Type B profiles and the differences were partially attributable to individual bird differences, rather than primarily differences in Hb saturation profile group. However, the Hb saturation increase to peak venous values in Type A profiles was more than 5 times greater than that in Type B dives and driven by saturation profile group, not individual differences. This increase in Hb saturation was also over 4 times greater than that in the aorta, resulting in Type A saturation levels approaching arterial. Further, the Type A peak saturation (94.1%) was much greater than the 72% saturation calculated on the basis of measured arterial and venous O_2_ content at rest and also greater than the 78% value estimated with a venous blood O_2_ content calculation of arterial O_2_ content at 99% saturation – 5 ml O_2_ dl^−1^ (Kooyman and Ponganis, 1998; Ponganis et al., 2007). Such a large increase in venous saturation is most consistent with decreased tissue O_2_ uptake and probably utilization of a-v shunting. The elevated heart rates that occur during this phase of the dive (Meir et al., 2008; Wright et al., 2014) could provide the blood flow to support such shunting. Because arterial blood is oxygenated and stroke effort is highest during this phase of the dive (Meir et al., 2008; van Dam et al., 2002; Williams et al., 2012), hypometabolism secondary to hypoxia or decreased locomotory effort seems unlikely. Rather, decreased regional tissue perfusion and a-v shunting appear more plausible. The lack of a significant difference in mean dive Hb saturation between Type A venous profiles and arterial profiles also supports the use of a-v shunting.

Decreases in Type A venous Hb saturation profiles were slow, often less than 10% in magnitude, and, similar to arterial saturation profiles, only occurred more rapidly during late ascent. Type A end-of-dive Hb saturation was also not significantly different from the corresponding arterial value. These similarities argue that a-v shunting may continue throughout most if not all of a dive with a Type A profile, and that end-of-dive decreases in arterial Hb saturation contribute significantly to the corresponding declines in venous Hb saturation. A-v shunting during a dive would have implications not only for O_2_ store management, but also for nitrogen uptake/distribution during dives (Fahlman et al., 2007). The relatively high arterial and Type A venous end-of-dive Hb saturation even in dives beyond the ADL also supports the concept that the rise in post-dive blood lactate concentration at the ADL is primarily due to depletion of the muscle O_2_ store and the onset of muscle lactate accumulation during the dive (Williams et al., 2011). Although depletion of the respiratory and blood O_2_ stores is highly variable, these O_2_ depots are typically not completely depleted at the ADL and/or in many dives beyond the ADL (Meir and Ponganis, 2009; Stockard et al., 2005; Williams et al., 2021).

#### Type B Hb saturation profiles are consistent with Type B Mb saturation profiles

Type B venous Hb saturation profiles were dominated by an overall progressive decline in saturation, which is consistent with the slower saturation decline in Type B Mb saturation profiles (Figs 2 and 3). Saturation sometimes increased at the start of the dive but, often, the initial saturation was the peak value. The average increase in peak Hb saturation in Type B profiles was about one-fifth that in Type A profiles. The rate of decline of Hb saturation from peak values and the magnitude of decline between peak and end-of-dive saturations in Type B profiles were also faster and greater than those in Type A venous saturation profiles. All this evidence supports the hypothesis that there is greater tissue perfusion, blood O_2_ extraction and blood O_2_ supplementation of muscle metabolism in dives with Type B venous Hb saturation profiles, consistent with Type B Mb saturation profiles.

The transient but sometimes prolonged interruptions in the Type B venous Hb saturation profiles are also consistent with Type B muscle Mb desaturation profiles observed by Williams et al. (2011) and with the hypothesis that muscle blood flow can be variable during a dive. In Type B Mb saturation profiles, the decline in Mb saturation was slow, could plateau mid-dive, and often progressed more rapidly and completely during the latter segments of long dives. Those findings in muscle are consistent with the predominant decreasing venous Hb saturation profiles and the interruption points in venous Hb saturation observed in Type B venous profiles in this study (Figs 4 and 6B).

#### Transient interruptions in Type B profiles further support the plasticity of the dive response and a-v shunting

Although large declines in Hb saturation dominated Type B venous profiles, the progressive declines were often interrupted by transient changes (increases and/or plateaus) in venous saturation in 98% of dives with Type B profiles (Fig. 4). Transient interruptions were usually associated with a change in vertical direction, and could occur during descent and ascent, and at maximum depth. We propose that such interruptions in venous Hb desaturation are secondary to changes in peripheral vascular regulation and/or heart rate, and represent times where tissue O_2_ uptake is decreased by regional vasoconstriction and/or by utilization of a-v shunts. The neuroregulatory mechanism responsible for such changes in these cardiovascular responses is unclear.

As illustrated in the 9.9 min dive of one emperor penguin (EP 17; Fig. 6B), the prolonged inflection in the latter half of the saturation profile included a large increase and then decline in venous Hb saturation. We hypothesize that this inflection is secondary to vasoconstriction of tissues (including muscle) and simultaneous a-v shunting. The end result would be a large increase in venous Hb saturation during ascent that only declines in the final ascent as a result of the decline in arterial saturation (Fig. 6B). In other words, during this phase of the dive, peripheral vasoconstriction isolates muscle and/or other tissues from blood, while simultaneous a-v shunting delivers more oxygenated blood to the vein, but, in the final phase of the dive as arterial blood desaturates, so too does the venous blood.

#### A-v blood O_2_ differences further support the plasticity of the dive response

**Fig. 7.**
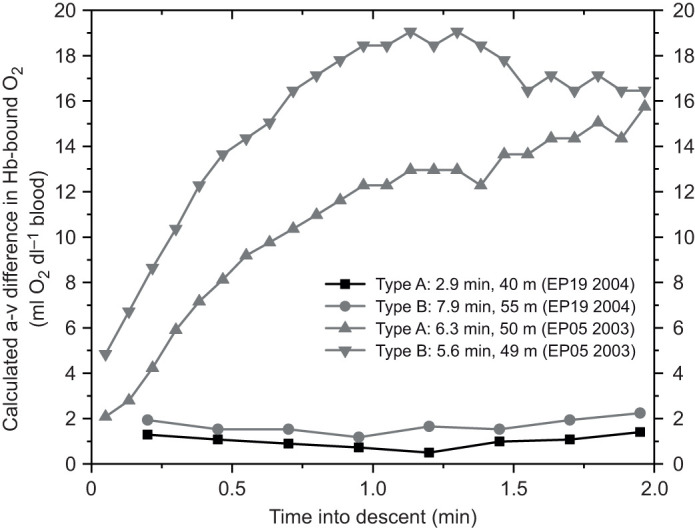
**Calculated arterio-venous (a-v) differences in Hb-bound O_2_ during the first 2** **min of a dive with a Type A venous Hb saturation profile and three dives with Type B venous Hb saturation profiles, demonstrating the range of blood O_2_ extraction during early descent.** Dives with Type A profiles were characterized by minimal blood O_2_ extraction while dives with Type B profiles had variable blood O_2_ extraction during early descent. The figure is based on venous Hb saturation profiles selected from Fig. 4B,C, an assumed arterial Hb saturation of 95% during this phase of the dive (see Fig. 1), a Hb concentration of 18 g dl^−1^, and 1.34 ml O_2_ g^−1^ Hb at full saturation.

The constant arterial Hb saturation profile during most of a dive's duration provides a valuable reference for interpretation of venous Hb saturation and calculation of a-v O_2_ difference estimates. The venous Hb saturation profiles and estimated a-v O_2_ content differences can serve as an index of tissue blood flow and the magnitude of blood O_2_ extraction by tissues. It is notable that during the earlier portions of dives, times at which arterial Hb saturation is typically 95–100%, there was a wide range of venous Hb saturation and calculated a-v differences in Hb-bound O_2_ content (Figs 6A,B and 7). Dives with Type A venous profiles typically had very small a-v O_2_ differences, consistent with Type A Mb–O_2_ profiles, peripheral vasoconstriction, minimal blood O_2_ extraction by tissue and possible a-v shunting (Figs 4A and 7).

In contrast, Type B venous Hb saturation profiles, in general, had a wide range of saturations during the first 2 min of dives, consistent with the Type B Mb desaturation profiles in Williams et al. (2011) and supportive of the concept of a spectrum of cardiovascular responses and muscle blood flow patterns during dives with Type B venous profiles (Fig. 4). Small a-v O_2_ differences during early descent occurred in some Type B profiles (Fig. 7), consistent with our hypotheses of variable muscle blood flow and O_2_ extraction patterns in dives with Type B venous profiles.

In other Type B venous profiles, Hb saturation during descent was as low as 10–40% (Fig. 4). In these types of dives, the calculated a-v differences in Hb-bound O_2_, based on an arterial saturation of 95% and venous saturation profiles during the first 2 min of a dive, were as high as 19 ml O_2_ dl^−1^ (Fig. 7), almost 4 times the measured value in emperor penguins at rest (Ponganis et al., 2007). In comparison, the a-v O_2_ content differences in flying pigeons, running emus and running bar-headed geese, all exercising at high intensity, were about 10 ml O_2_ dl^−1^ (Butler et al., 1977; Grubb et al., 1983; Hawkes et al., 2014). In the running dog at maximum O_2_ consumption, the a-v O_2_ difference was 15 ml O_2_ dl^−1^ (Taylor et al., 1987). Given the low average locomotory muscle oxygen consumption (12.4 ml O_2_ kg^−1^ muscle min^−1^) during dives (Williams et al., 2011), the extremely low venous Hb saturation and large a-v O_2_ differences during these particular Type B dives suggest (a) muscle oxygen consumption (stroke rate, work effort) may have been higher than average, (b) vasoconstriction may have been less and muscle blood flow greater than average, and/or (c) Mb may not have been completely saturated prior to diving (see Scholander et al., 1942). In addition, the possibility of anemia (low Hb content) or unusually low arterial Hb saturation in these particular birds cannot be ruled out. Clearly, simultaneous measurement of arterial and venous Hb saturation profiles and venous Hb and muscle Mb saturation profiles and stroke effort is desirable. Nonetheless, the broad range of calculated a-v O_2_ differences and venous Hb desaturation profiles observed within and among different dives were consistent with the Mb desaturation profiles of Williams et al. (2011) and the wide end-of-dive range in air sac and blood O_2_ content (Meir and Ponganis, 2009; Stockard et al., 2005; Williams et al., 2021). All these findings support the concept of a spectrum of both heart rate and peripheral vascular responses in dives of emperor penguins.

### Conclusions

On the basis of Mb saturation profiles and heart rate profiles, both the cardiac and peripheral vascular components of the dive response have been considered quite ‘plastic’ and dependent on the nature of a given dive in emperor penguins (Meir et al., 2008; Williams et al., 2011; Wright et al., 2014). The venous saturation profiles in this study support this concept of plasticity in the dive response, including not only heart rate but also the peripheral vascular component. Accordingly, although there are distinct differences, we emphasize that the dive response and resulting Hb and Mb saturation profiles in emperor penguins cannot even be simply characterized into only Types A and B. Rather, the dive response is a spectrum of cardiac and vascular components dependent on the nature and demands of a given dive and even of a given segment of a dive.

## Supplementary Material

10.1242/jexbio.246832_sup1 Supplementary information
