# Supplementary material for: Blood oxygen transport and depletion in diving emperor penguins
Source: J Exp Biol. 2024 Mar 18;227(6):jeb246832. doi: 10.1242/jeb.246832 (PMC11006389; doi:10.1242/jeb.246832)
Supplement: Supplementary information [file jexbio-227-246832-s1.pdf]

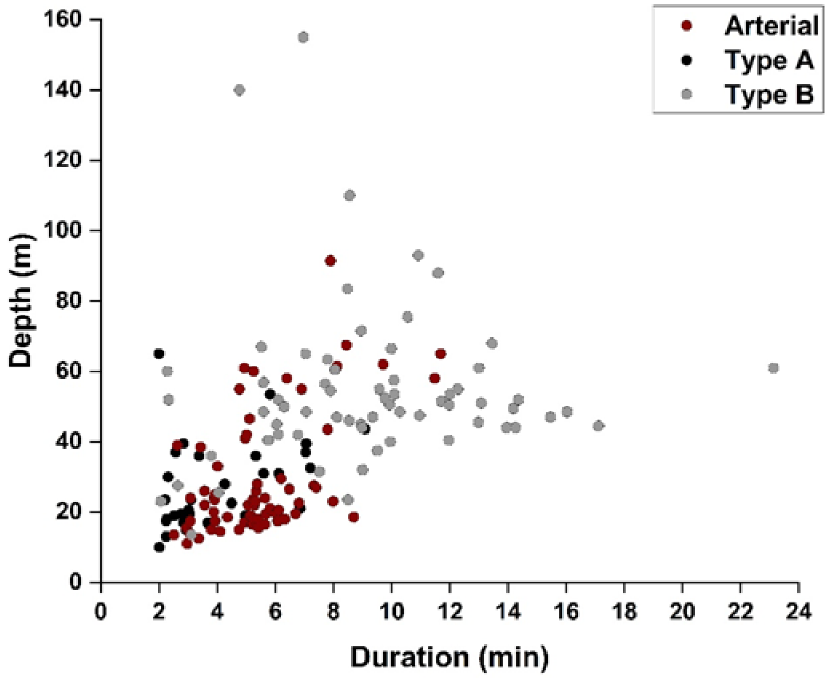

Fig. S1. Dive depth compared with dive duration for dives in the three hemoglobin saturation profile groups: Arterial saturation profile (Arterial, maroon), Type A venous saturation profile (Type A, black) and Type B venous saturation profile (Type B, gray). For all dives, depth had a significant effect on duration ($X^2(1)=10.13$, $p<0.01$). n= 15 penguins, 160 dives.

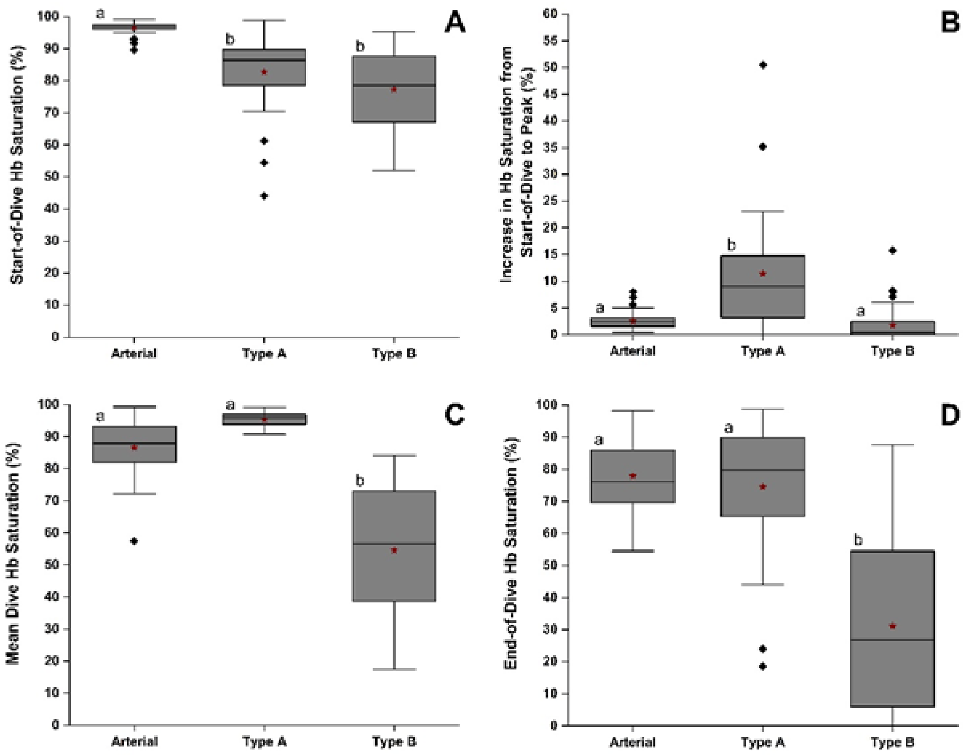

Fig. S2. Box plot charts showing significant differences among different types of dives (arterial, Type A venous, and Type B venous) for the start-of-dive hemoglobin (Hb) saturation (A), the increase in Hb saturation from the start of dive to the maximum Hb saturation (B), the mean Hb saturation for the dive (C), and the end-of-dive Hb saturation (D). The letters above box plots represent the results of pairwise comparisons using Tukey's method. Within each panel, different letters represent a significant difference between the dive types. N = 15 penguins, 160 dives for all analyses. The gray box (▭) indicates the upper and lower quartiles and includes the median line. Whiskers (⊥) extend to 1.5x the interquartile range and outliers are indicated by diamonds (♦). Mean values are represented by a red star (★).

Table S1. Year of measurements, penguin identification (ID), number of dives used in analyses, mean and maximum durations of dives in minutes (min), type of oxygen (O_2) sampling site (arterial or venous), and number of Type A or Type B dives identified.

Year	Penguin ID	# Dives	Duration (min) (mean, max)	O_2 Sampling Site	# Type A	# Type B
2001	37	9	5.1, 8.6	Venous	2	7
2001	36A	6	4.4, 5.3	Arterial	NA	NA
2003	2	1	3.1, 3.1	Arterial	NA	NA
2003	4	5	6.1, 6.9	Venous	5	0
2003	5	11	5.0, 9.1	Venous	5	6
2003	7	10	5.3, 7.2	Venous	10	0
2004	17	14	6.0, 16.0	Venous	4	10
2004	19	19	10.9, 23.1	Venous	4	15
2004	20	3	7.1, 10.3	Venous	1	2
2005	36	5	10.5, 13.0	Venous	0	5
2005	40	2	11.3, 12.0	Venous	0	2
2005	48	18	9.3, 13.1	Venous	0	18
2007	8	12	5.0, 8.7	Arterial	NA	NA
2007	10	32	5.2, 8.0	Arterial	NA	NA
2008	1	13	7.4, 11.7	Arterial	NA	NA

Journal of Experimental Biology • Supplementary information
